# Hyperoside alleviates adriamycin-induced podocyte injury via inhibiting mitochondrial fission

**DOI:** 10.18632/oncotarget.21287

**Published:** 2017-09-28

**Authors:** Zhuyun Chen, Xiaofei An, Xi Liu, Jia Qi, Dafa Ding, Min Zhao, Suyan Duan, Zhimin Huang, Chengning Zhang, Lin Wu, Bo Zhang, Aihua Zhang, Yanggang Yuan, Changying Xing

**Affiliations:** ^1^ Department of Nephrology, The First Affiliated Hospital of Nanjing Medical University, Nanjing Medical University, Nanjing, China; ^2^ Department of Endocrinology, Jiangsu Province Hospital of Chinese Medicine, Affiliated Hospital of Nanjing University of Chinese Medicine, Nanjing, China; ^3^ Department of Pharmacy, Xinhua Hospital Affiliated to Shanghai Jiaotong University School of Medicine, Shanghai, China; ^4^ Department of Endocrinology, The Second Affiliated Hospital of Nanjing Medical University, Nanjing Medical University, Nanjing, China; ^5^ Department of Nephrology, Nanjing Children’s Hospital, Nanjing Medical University, Nanjing, China

**Keywords:** hyperoside, adriamycin, mitochondrial fission, podocyte injury

## Abstract

Podocyte injury underlies many forms of glomerular diseases. Our previous study showed that hyperoside, a naturally occurring flavonoid, could decrease albuminuria at the early stage of diabetic nephropathy by ameliorating renal damage and podocyte injury. However, its protective mechanism against podocyte injury is unknown. A previous study demonstrated that hyperoside might inhibit amyloid β-protein-induced neurotoxicity by suppressing mitochondrial dysfunction. Both mitochondrial dysfunction and its upstream determinant mitochondrial fission were closely related to podocyte injury. Thus, in the current study, we tested the effect of hyperoside on mitochondrial dysfunction and mitochondrial fission in adriamycin (ADR)-induced podocyte injury. In the mice model of ADR-induced nephropathy, hyperoside treatment inhibited ADR-induced albuminuria and podocyte injury. Meanwhile, hyperoside also blocked ADR-induced mitochondrial dysfunction and mitochondrial fission. Consistently, in cultured human podocytes, hyperoside suppressed ADR-induced podocyte injury, mitochondrial dysfunction and mitochondrial fission. All these results indicated that hyperoside might inhibit ADR-induced mitochondrial dysfunction and podocyte injury through suppressing mitochondrial fission both *in vivo* and *in vitro*. The underlying mechanisms which we revealed support the therapeutic effects of hyperoside for a broad range of glomerular diseases.

## INTRODUCTION

Podocytes, which are highly specialized, terminally differentiated epithelial cells lining the outer surface of the glomerular capillaries, serve as a critical size and charge barrier to prevent proteinuria [1]. Independent laboratories have reported that podocyte injury plays an essential role in causing the defective glomerular filtrations and the onset of proteinuria [2]. Regardless of the diverse origins of glomerular diseases, podocytes are critical determinants in the progression of all glomerular diseases [3]. Therefore, exploration of pathogenic mechanisms of podocyte injury is important for the future treatment. Our previous study demonstrated that mitochondrial dysfunction mediated aldosterone-induced podocyte injury [4]. Thus, targeting mitochondria may offer a novel approach to reduce podocyte injury.

The many functions of mitochondria have been intimately connected to their morphology, which is a delicate balance between the fusion and fission [5]. Both mitochondrial fusion and fission contribute to maintenance and optimization of mitochondrial function [6]. In cultured muscle cells, inhibition of mitochondrial fission protected against palmitate-induced mitochondrial dysfunction and insulin resistance [7]. It was demonstrated that mitochondrial fission was an important factor resulting in podocyte injury [8]. Our recent study found that inhibition of the mitochondrial fission protein dynamin-related protein 1 (Drp1) suppressed aldosterone-induced mitochondrial dysfunction and podocyte injury [9]. Therefore, the blockage of mitochondrial dysfunction by suppressing mitochondrial fission could hold the key to potential treatment for podocyte injury.

Flavonoids have many beneficial effects including inhibiting carcinogenesis, reducing the contents of low-density lipoprotein, lowering hypertension and decreasing the generation of reactive oxygen species (ROS) [10]. Hyperoside, as a naturally occurring flavonoid, has been documented as having anti-inflammatory, anti-oxidation anti-apoptosis and diuretic properties [11]. It was reported that a combination of quercetin and hyperoside prevented unilateral ureteral obstruction-induced renal fibrosis [12]. Our previous study showed that hyperoside could decrease albuminuria at the early stage of diabetic nephropathy by ameliorating renal damage and podocyte injury [13]. However, its renoprotective mechanism is still unclear. A previous study revealed that hyperoside might inhibit amyloid β-protein-induced neurotoxicity by suppressing mitochondrial dysfunction [14]. Until now, the role of hyperoside on mitochondrial function and its determinant mitochondrial morphology in podocyte injury was not investigated.

Thus, to elucidate this, in the current study we utilized the murine model of adriamycin (ADR) nephropathy, which recapitulates the human disease of focal segmental glomerulosclerosis (FSGS) showing initial podocyte injury and albuminuria and subsequent renal fibrosis [15]. We found that hyperoside might inhibit adriamycin-induced mitochondrial dysfunction and podocyte injury via regulating mitochondrial fission. These results were subsequently confirmed by *in vitro* study by using cultured human podocytes. The underlying mechanisms which we revealed support the therapeutic effects of hyperoside for a broad range of glomerular diseases.

## RESULTS

### Hyperoside prevented renal structural changes and albuminuria in adriamycin mice

**Figure 1:**
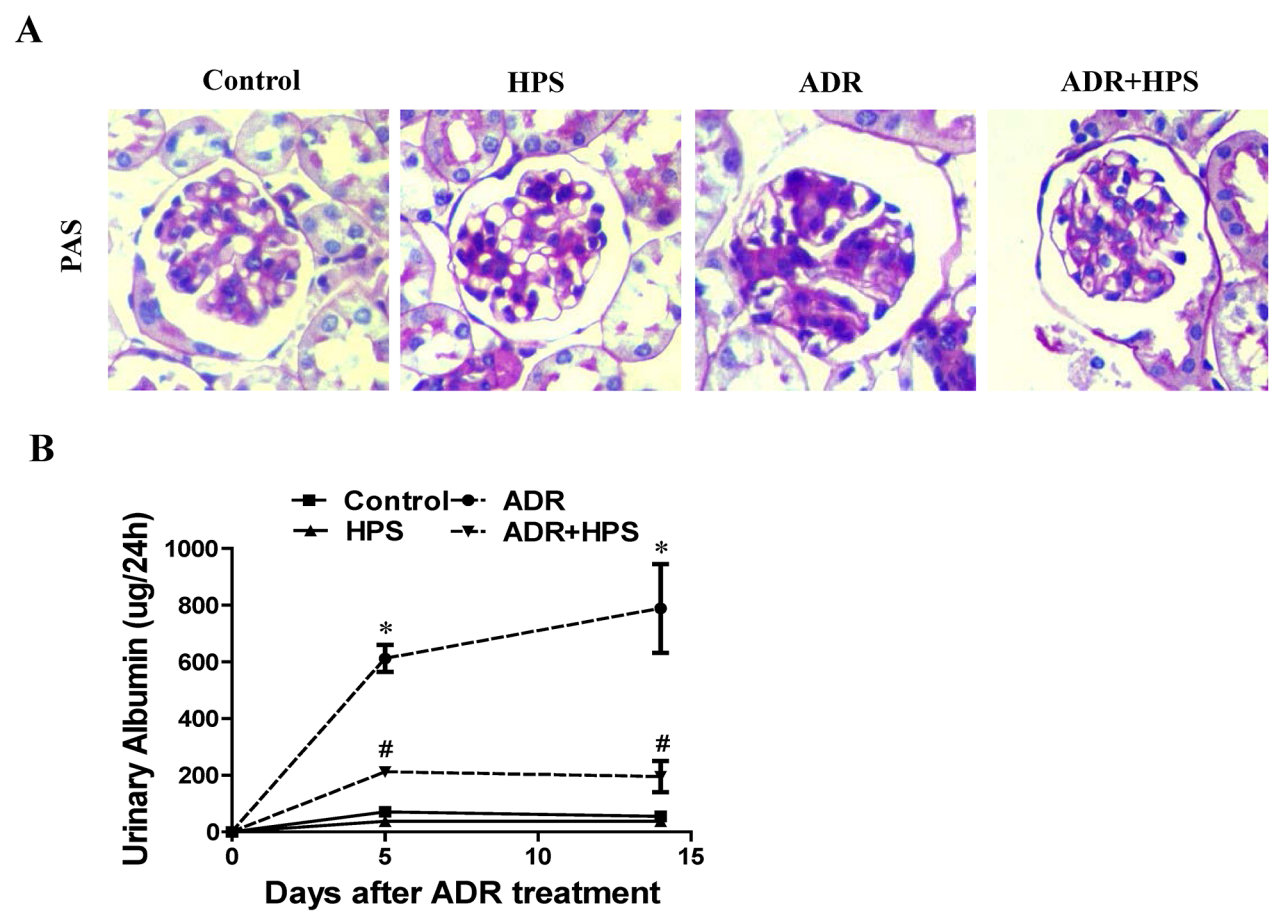
Effect of hyperoside on adriamycin-induced kidney injury and albuminuria *in vivo* **(A)** Kidney histology (×400). **(B)** Urinary albumin levels. Values are means ± SEM (n=6 for each group). *P <0.05 vs. control group, ^#^*P*<0.05 vs. ADR group. ADR, adriamycin; HPS, hyperoside.

To determine the effect of hyperoside in adriamycin nephropathy, we examined the kidneys of these mice histopathologically. As shown in Figure 1A, morphometric analysis of kidney histology revealed an increase in mesangial matrix area in ADR kidneys as compared with control kidneys. Treatment of hyperoside inhibited the glomerular mesangial cell proliferation by ADR. Moreover, albuminuria was reduced by hyperoside treatment in ADR mice (Figure 1B). These data indicated that hyperoside reduced ADR-induced kidney injury and albuminuria.

### Hyperoside inhibited podocyte injury in adriamycin mice

**Figure 2:**
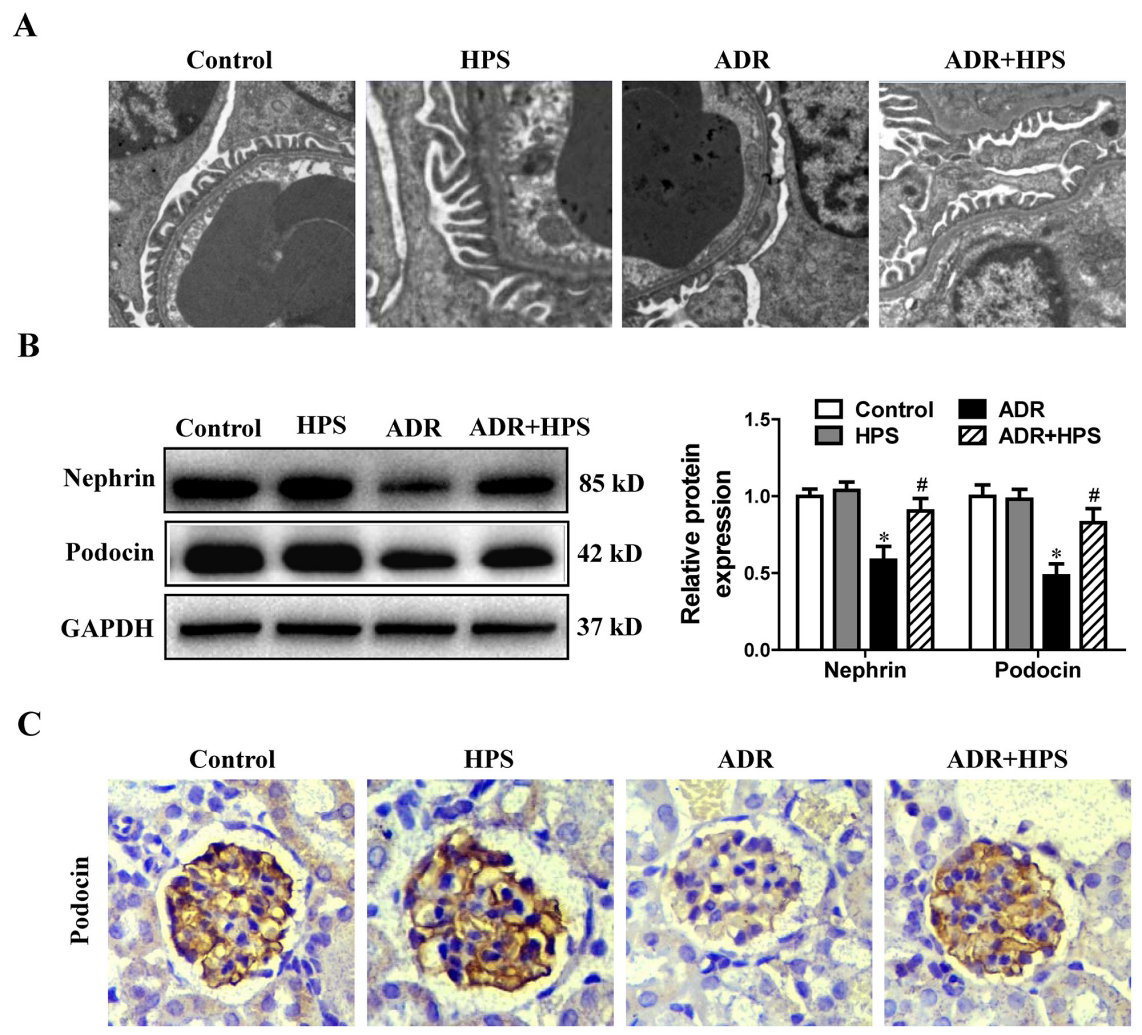
Effect of hyperoside on adriamycin-induced podocyte injury *in vivo* **(A)** Foot processes of podocytes by transmission electron microscopy. **(B)** Western blots for nephrin and podocin expressions. Left: representative immunoblots. Right: densitometric analysis. **(C)** Immunohistochemical staining of glomerulus by podocin (Magnification×400). Values are means ± SEM (n=6 for each group). *P <0.05 vs. control group, ^#^*P*<0.05 vs. ADR group. ADR, adriamycin; HPS, hyperoside.

To determine the role of hyperoside on podocyte injury in ADR mice, we observed podocytes foot process structure and examined the expression of podocyte-specific proteins nephrin and podocin. In electron micrographs, the extensive fusion of foot processes was evident in ADR mice. Treatment of the ADR mice with hyperoside maintained the normal shape of the foot processes (Figure 2A). Additionally, hyperoside restored the protein levels of both nephrin and podocin (Figure 2B). Immunohistochemistry staining for podocin also showed hyperoside enhanced the expression of podocin which was decreased under ADR injection (Figure 2C). These data indicated that hyperoside suppressed ADR-induced podocyte injury.

### Hyperoside suppressed mitochondrial dysfunction in adriamycin mice

**Figure 3:**
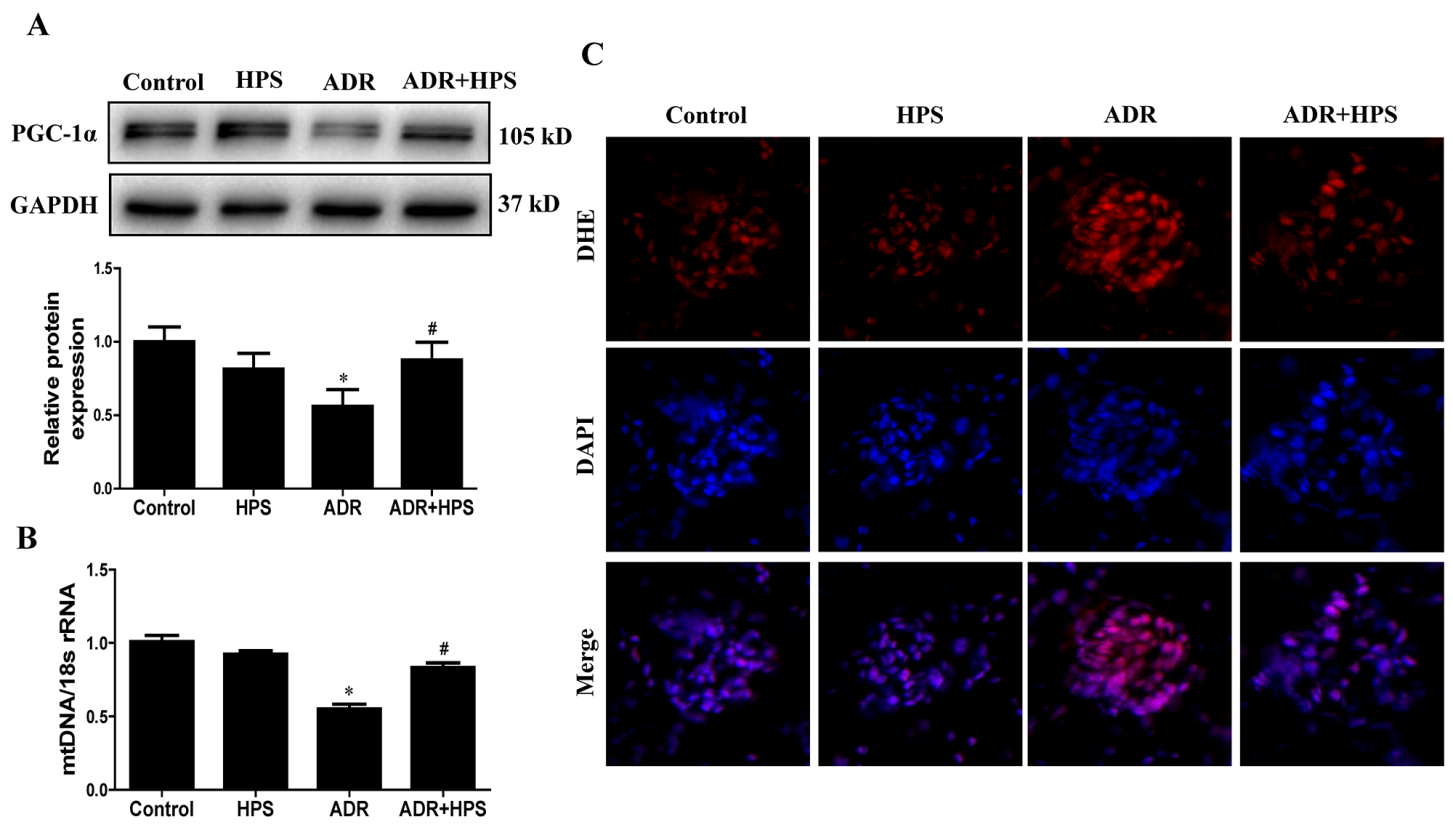
Effect of hyperoside on adriamycin-induced mitochondrial dysfunction *in vivo* **(A)** PGC-1α protein expression was detected by immunoblotting. Upper: representative immunoblots. Lower: densitometric analysis. **(B)** mitochondrial DNA (mtDNA) copy number detected by real-time PCR. **(C)** Representative images of DHE staining to detect ROS (red) and DAPI staining to detect nuclei (blue). Magnification ×400. Values are means ± SEM (n=6 for each group). *P <0.05 vs. control group, ^#^*P*<0.05 vs. ADR group. ADR, adriamycin; HPS, hyperoside.

To evaluate mitochondrial dysfunction *in vivo*, we used several independent parameters: peroxisome-proliferator-activated receptor γ co-activator-1α (PGC-1α) expression, mtDNA copy number and reactive oxygen species (ROS) production. PGC-1α is a central regulator of mitochondrial biogenesis [16]. Our previous work demonstrated that downregulation of PGC-1α expression in podocyte could induce mitochondrial dysfunction [17]. As shown in Figure 3A, PGC-1α expression was inhibited in ADR mice, and hyperoside restored PGC-1α protein levels. Mitochondrial function is under the dual control of nuclear and mitochondrial DNA [18]. Maintaining mtDNA copy number is critical for preserving mitochondrial function [19]. Hyperoside treatment also rescued the mtDNA copy number in ADR mice (Figure 3B). Mitochondria are the primary sources of ROS generation [20]. Then, we assessed ROS production in the kidneys through DHE staining. As shown in Figure 3C, hyperoside inhibited ADR induced the ROS production in glomeruli. Thus, hyperoside treatment could inhibit ADR-induced mitochondrial dysfunction.

### Hyperoside attenuated mitochondrial fission in adriamycin mice

**Figure 4:**
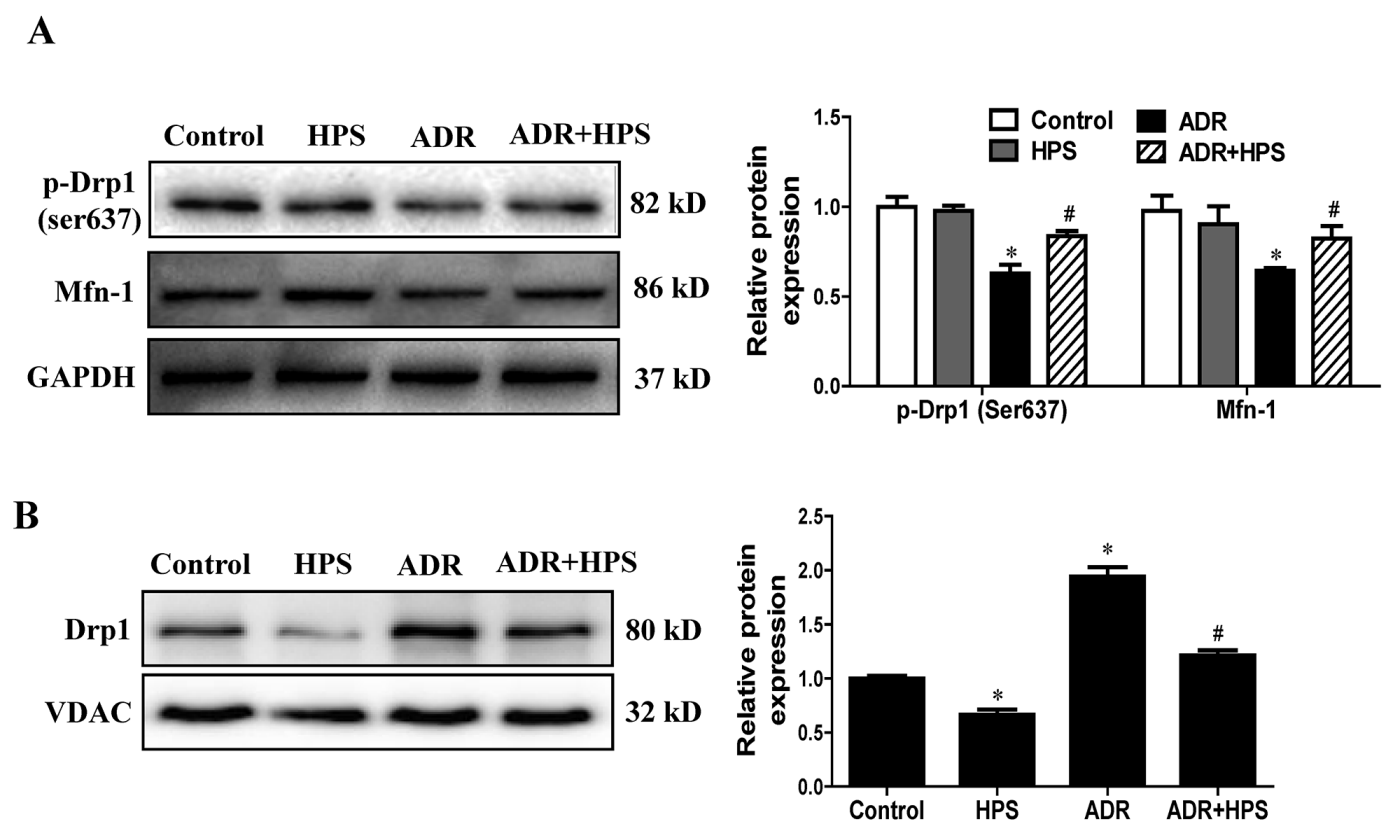
Effect of hyperoside on adriamycin-induced mitochondrial fission *in vivo* **(A)** Western blots for Drp1 phosphorylation on ser637 and Mfn-1. GAPDH serves as a loading control. Left: representative immunoblots. Right: densitometric analysis. **(B)** Drp1 expression in mitochondrial fraction was evaluated using Western blot analysis. Left: representative immunoblots. Right: densitometric analysis. Values are means ± SEM (n=6 for each group). *P <0.05 vs. control group, ^#^*P*<0.05 vs. ADR group. ADR, adriamycin; HPS, hyperoside.

Given that mitochondrial dysfunction could be mediated by mitochondrial fission, we then detected the effects of hyperoside on mitochondrial fission. The cytoplasmic dynamin-related GTPase Drp1 is a key mediator of mitochondrial fission, while fusion proteins Mfn-1 and Mfn-2 are essential for fusion reaction [21]. Dephosphorylation of Drp1 at serine-637 triggers the translocation of Drp1 from the cytoplasm to mitochondria and results in the fragmentation of mitochondria [22]. As expected, ADR decreased the phosphorylation of Drp1 at serine 637 and reduced the expression of Mfn-1, which were blocked by hyperoside treatment (Figure 4A). Furthermore, in the mitochondrial fraction, we found that Drp1 increased significantly in the ADR group compared with the control group, which was also blocked by hyperoside (Figure 4B). Therefore, these results suggested that hyperoside inhibited ADR-induced mitochondrial fission.

### Hyperoside inhibited adriamycin-induced podocyte injury *in vitro*

**Figure 5:**
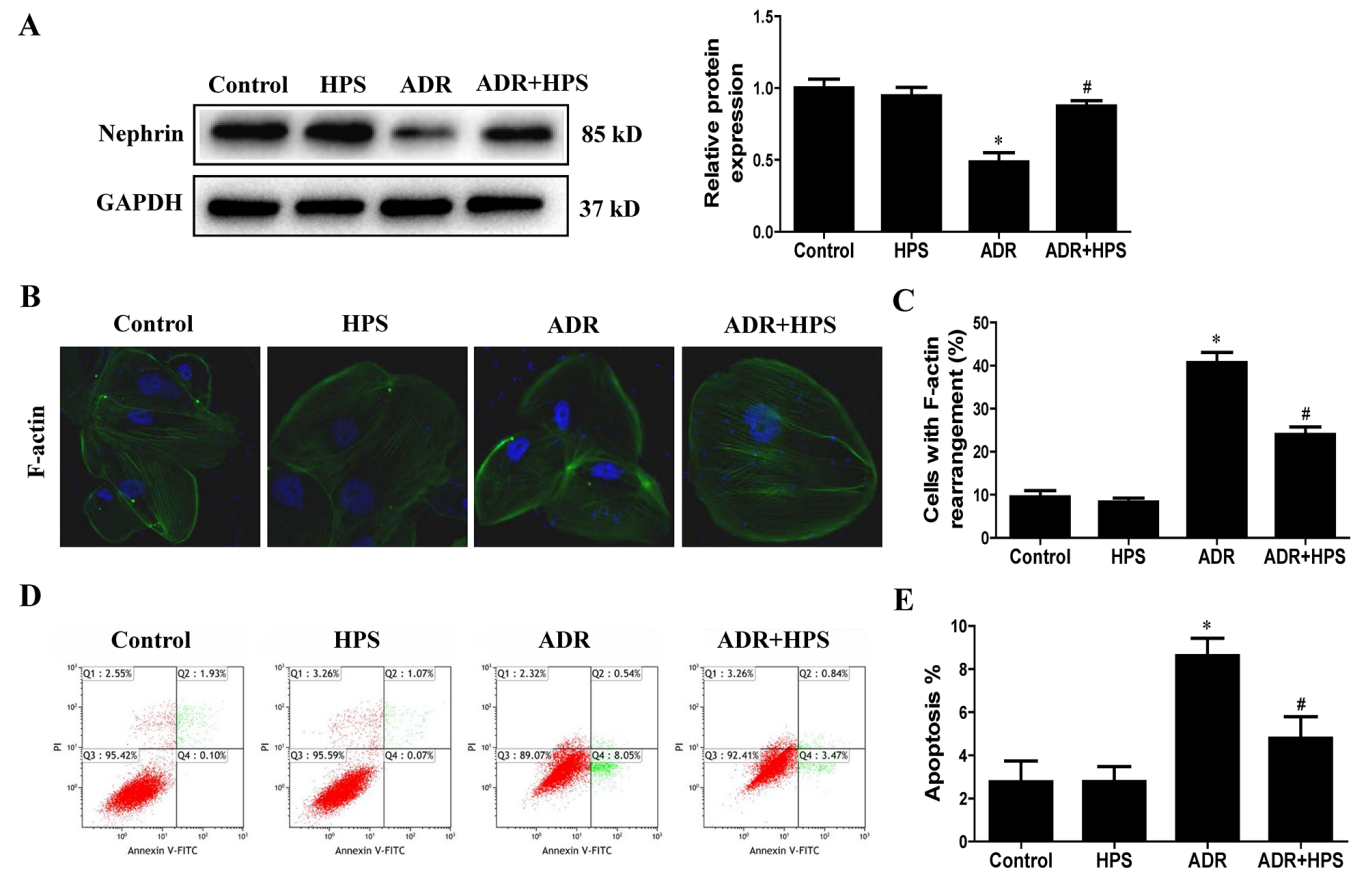
Effect of hyperoside on adriamycin-induced podocyte injury *in vitro* Podocytes were pre-treated with hyperoside (50μmol/L) for 1 h followed by co-incubation with ADR (1μg/ml) for further 12 h. **(A)** Western blots for nephrin expression. Left: representative immunoblots. Right: densitometric analysis. **(B)** F-actin was detected by phalloidin fluorescence labelling in cultured podocytes. **(C)** Quantification of cells with F-actin rearrangement. **(D)** Representative pictures of apoptosis determined by flow cytometry. **(E)** Quantification of apoptosis by flow cytometry. Values are means ± SEM from three independent experiments. *P <0.05 vs. control group, ^#^*P*<0.05 vs. ADR group. ADR, adriamycin; HPS, hyperoside.

In cultured podocytes, the nephrin expression was significantly decreased when exposed to ADR. Hyperoside treatment restored nephrin expression (Figure 5A). In addition, hyperoside ameliorated the ADR-induced disruption of F-actin filament (Figure 5B and 5C). Moreover, hyperoside prevented ADR-induced podocyte apoptosis as assessed using annexin V/flow cytometry detection (Figure 5D and 5E). These data indicated hyperoside inhibited ADR-induced podocyte injury *in vitro*.

### Hyperoside inhibited adriamycin-induced mitochondrial dysfunction *in vitro*

**Figure 6:**
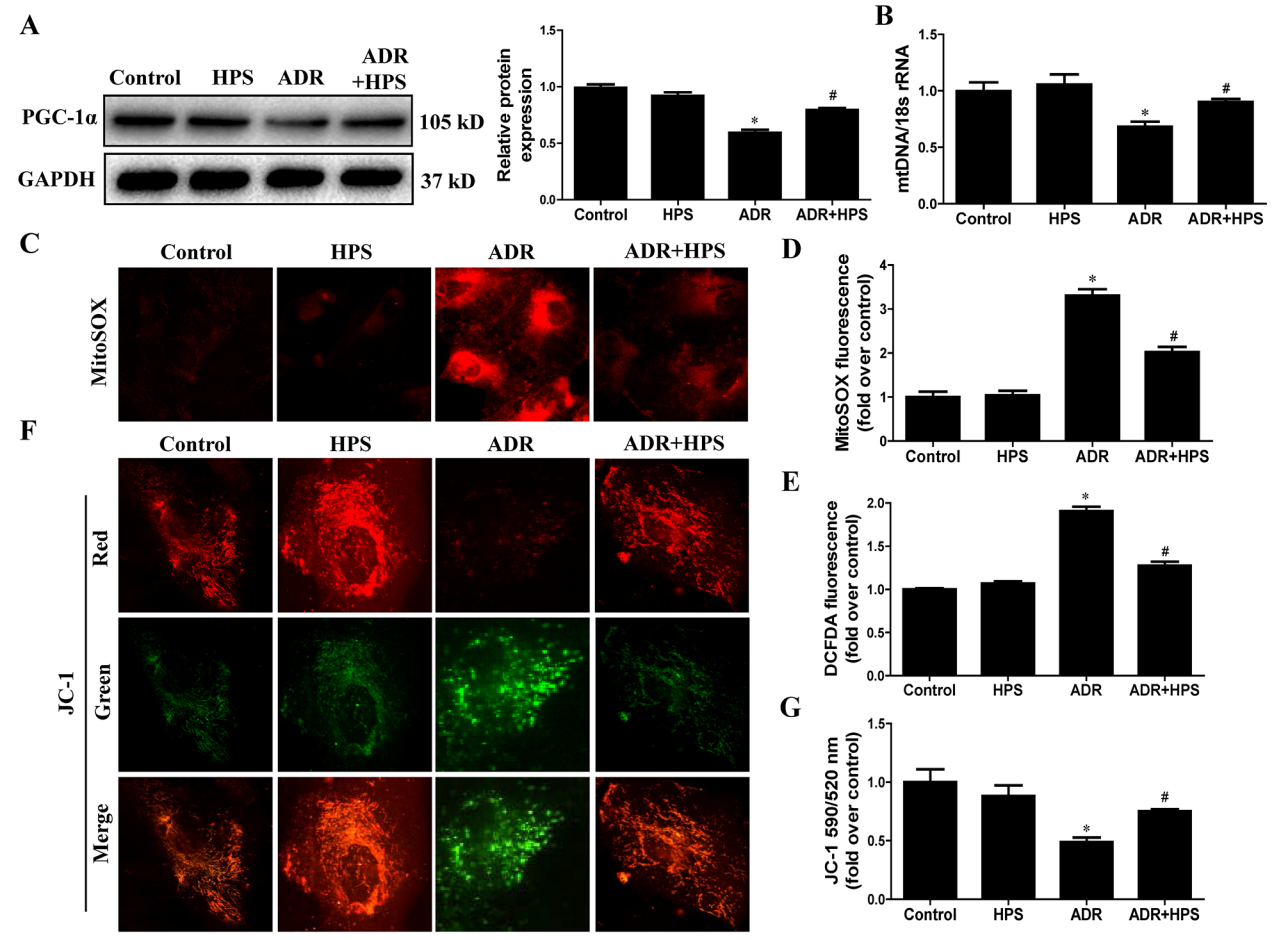
Effect of hyperoside on adriamycin-induced mitochondrial dysfunction *in vitro* Podocytes were pre-treated with hyperoside (50μmol/L) for 1 h followed by co-incubation with ADR (1μg/ml) for further 12 h. **(A)** Western blots for PGC-1α expression. Left: representative immunoblots. Right: densitometric analysis. **(B)** mtDNA copy number. **(C)** Representative images of podocytes stained with MitoSOX. **(D)** Quantitation of MitoSOX by flow cytometry. **(E)** Podocytes were stained with DCFDA and the DCF fluorescence intensities were analyzed by flow cytometry. **(F)** Representative images of podocytes stained with JC-1. **(G)** Quantitation of mitochondrial membrane potential changes by flow cytometry. Values are means ± SEM from three independent experiments. *P <0.05 vs. control group, ^#^*P*<0.05 vs. ADR group. ADR, adriamycin; HPS, hyperoside.

Consistent with *in vivo* findings, we tested the effect of hyperoside on ADR-induced mitochondrial dysfunction in cultured podocytes. As shown in Figure 6A, hyperoside restored the expression of PGC-1α. Also, hyperoside treatment rescued the mtDNA copy number (Figure 6B). Additionally, as shown in Figure 6C and 6D, ADR-induced mitochondrial ROS generation stained by mitoSOX was abolished following pretreatment with hyperoside. Hyperoside also inhibited ADR-induced H_2_O_2_ production detected by DCFDA staining (Figure 6E). Meanwhile, JC-1 staining showed that hyperoside blocked the transition from red fluorescence to green fluorescence induced by ADR (Figure 6F and 6G), suggesting that hyperoside treatment restored mitochondrial membrane potential. Taken together, these studies indicated that hyperoside inhibited ADR-induced mitochondrial dysfunction *in vitro*.

### Hyperoside inhibited adriamycin-induced mitochondrial fission *in vitro*

**Figure 7:**
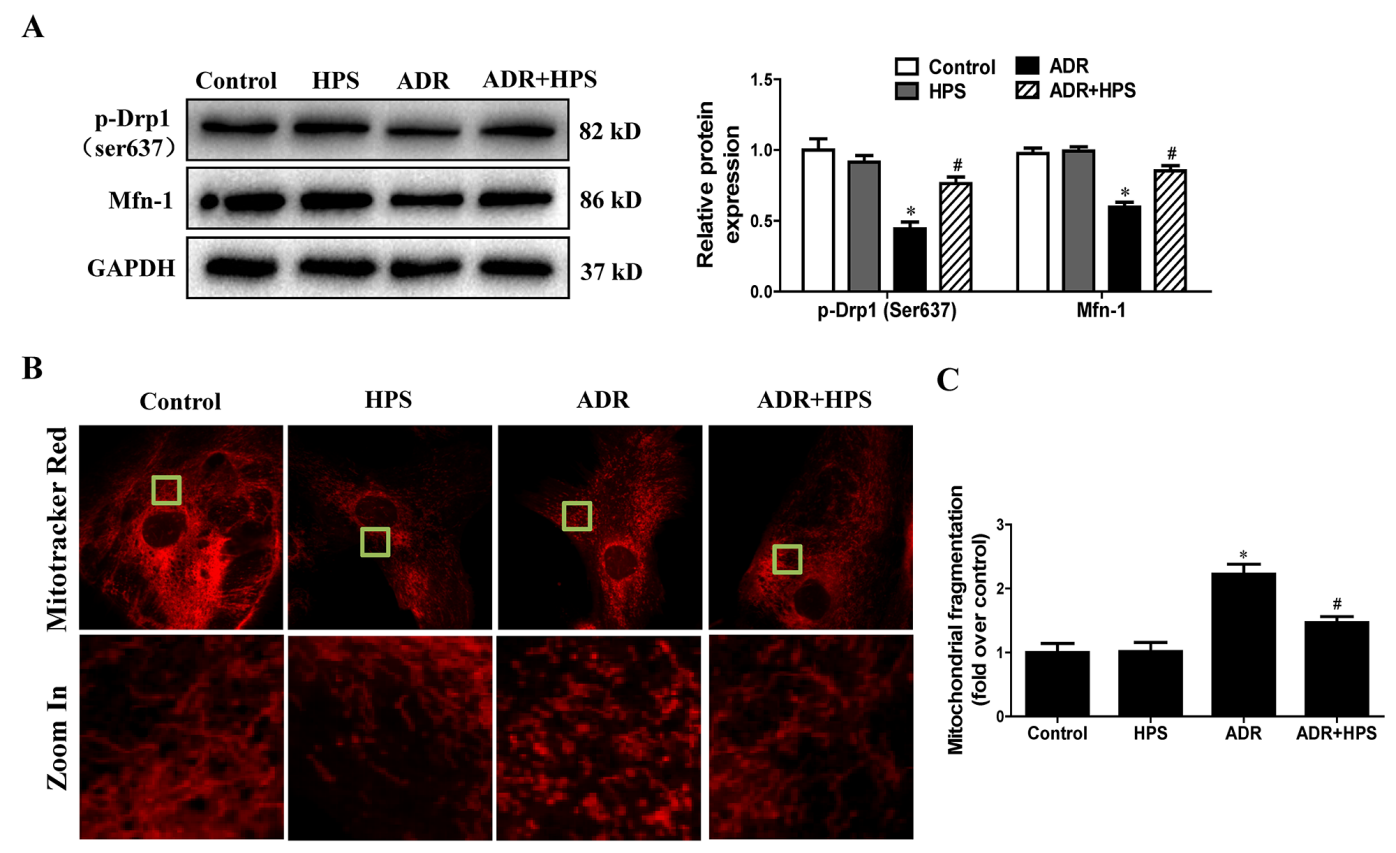
Effect of hyperoside on adriamycin-induced mitochondrial fission *in vitro* Podocytes were pre-treated with hyperoside (50μmol/L) for 1 h followed by co-incubation with ADR (1μg/ml) for further 12 h. **(A)** Western blots for Drp1 phosphorylation on ser637 and Mfn-1. Left: representative immunoblots. Right: densitometric analysis. **(B)** Micrographs of mitochondrial morphology visualized by MitoTracker Red staining of podocytes. **(C)** Quantification of mitochondrial morphology. Values are means ± SEM from three independent experiments. *P <0.05 vs. control group, ^#^*P*<0.05 vs. ADR group. ADR, adriamycin; HPS, hyperoside.

We further sought *in vitro* support for the effect of hyperoside on ADR-induced mitochondrial fission. As shown in Figure 7A, hyperoside blocked ADR-reduced phosphorylation of Drp1 at serine-637 and protein levels of Mfn-1. Moreover, hyperoside blocked ADR-induced mitochondrial fragmentation visualized by MitoTracker Red staining (Figure 7B and 7C). Thus, hyperoside inhibited ADR-induced mitochondrial fission *in vitro*.

## DISCUSSION

Adriamycin (ADR), an anthracycline chemotherapeutic agent, has been widely used in various cancer treatment regimens [23]. ADR causes dose-dependent toxicities in heart, liver and kidneys [24], which may limit its clinical use. ADR-induced nephropathy in rodents is the widely used experimental model of human primary FSGS and has been extensively studied [25]. Podocytes are the major cells involved in the development of FSGS [26]. ADR induced podocyte foot processes effacement, albuminuria, and glomerulosclerosis [27]. In our ADR mice model, we also found that ADR induced podocyte injury, displaying impaired expression of slit diaphragm proteins, podocyte foot processes effacement and proteinuria *in vivo*, and showing impaired expression of slit diaphragm proteins, disruption of the cytoskeleton and podocyte apoptosis. However, mechanism of ADR-induced podocyte injury is still unclear. The previous study has identified that mtDNA mutations and reduction in mtDNA copy number contributed to ADR-induced tissue injury. A mutation in the protein kinase, DNA-activated, catalytic polypeptide (Prkdc) gene led to mitochondrial DNA depletion in podocytes, and increased susceptibility to ADR nephropathy [28]. Moreover, it was reported that mitochondrial dysfunction might be an early event in ADR-induced podocyte injury and ADR induced mitochondrial morphological changed from large and ellipsoid shape to the small [29]. Consistently, our present results showed that ADR-induced mitochondrial dysfunction and mitochondrial fission both *in vivo* and *in vitro*.

A growing number of studies showed that mitochondrial dysfunction was closely related to podocyte injury [30]. Several indicators of mitochondrial dysfunction were investigated in our present study. The first indicator which we detected was PGC-1α. The core role of PGC-1α in mitochondrial bioenergetics and respiration is well recognized [31]. Our previous study demonstrated that the overexpression of PGC-1α in mice remarkably ameliorated aldosterone-induced mitochondrial dysfunction and podocyte injury [32]. Secondly, we examined mtDNA copy numbers. All mitochondrial respiratory chain complexes are partly encoded by the mtDNA except for complex II [33]. Recently, mtDNA copy number has been used as a surrogate measure of mitochondrial function. In a community-based cohort study, higher mtDNA copy number associated with lower risk of incident chronic kidney disease [34]. Thirdly, we measured mitochondrial ROS production. The mitochondrial electron transport chain contains several redox centers which are capable of producing ROS [35]. ROS overproduction has been correlated with a variety of podocyte injury models [36]. Our previous study showed that the inhibition of mitochondrial ROS by a mitochondrial-targeted antioxidant blocked puromycin aminonucleoside-induced podocyte injury [37]. Finally, we tested mitochondrial membrane potential *in vitro*. Mitochondrial membrane potential is important for maintaining the physiological function of mitochondrial respiratory chain to produce ATP [38]. Loss of mitochondrial membrane potential resulted in cytochrome c release from mitochondria to cytosol and triggered apoptosis [39]. Here, hyperoside reversed ADR-induced mitochondrial dysfunction by increasing the expression of PGC-1α, enhancing mtDNA copy numbers, inhibiting mitochondrial ROS production and restoring mitochondrial membrane potential.

Mitochondrial fission contributes to mitochondrial dysfunction. We previously found that inhibition of mitochondrial fission could ameliorate mitochondrial dysfunction not only in podocytes [9] but also in renal tubular epithelial cells [40]. Therefore, we tested the effect of hyperoside on mitochondrial fission and found that hyperoside could inhibit ADR-induced mitochondrial fission both *in vivo* and *in vitro*. Drp1 is a key regulator of mitochondrial fission. Previous study demonstrated that FK506 preserved mitochondrial morphology and myocardial function following ischemia-reperfusion injury by preventing dephosphorylation of Drp1 S637 and inhibiting Drp1 translocation to the mitochondria [41]. Consistently, our result showed that hyperoside blocked Drp1 translocation to mitochondria by regulating Drp1 S637 phosphorylation. On the other hand, we found that hyperoside restored the expression of Mfn-1. Mfn-1 was able to stimulate mitochondrial fusion [42]. Thus, hyperoside might regulate dynamics of fusion and fission to achieve mitochondrial homeostasis under stress.

In conclusion, our results demonstrated that hyperoside might inhibit ADR induced mitochondrial dysfunction and podocyte injury through regulating mitochondrial fission both *in vivo* and *in vitro*. These findings provide novel insight into the reno-protective effect of hyperoside as well as evidence in favor of its use in a wide range of podocytopathy treatment.

## MATERIALS AND METHODS

### Reagents and antibodies

Adriamycin (ADR, catalog number D1515) and dihydroethidium (DHE, catalog number D7008) were obtained from Sigma-Aldrich (St. Louis, MO). Hyperoside (HPS) was purchased Zelang Biological Technology Company (Nanjing, China). Anti-nephrin (catalog number ab58968), anti-podocin (catalog number ab50339), anti-PGC-1α (catalog number ab54481), phosphor anti-Drp1 (S637) (catalog number ab193216) and anti-VDAC (voltage-dependent anion channel, catalog number ab34726) antibodies were obtained from Abcam (Cambridge, MA, USA). Anti-Drp1 (catalog number sc-32898) and anti-Mfn-1 (catalog number sc-166644) antibodies were purchased from Santa Cruz (Santa Cruz, CA). anti-GAPDH was purchased from Sanying biotechnology (Wuhan, China, catalog number 10494-1-AP). All secondary antibodies for immunoblot analysis were from Zhongshan Golden Bridge Biotechnology (Beijing, China, catalog number ZB-2301). MitoSOX (catalog number M36008) and 2’,7’-dichlorofluorescein diacetate (DCFDA, catalog number C6827) were from Invitrogen (Carlsbad, CA, USA).

### Animals

8-week-old male Balb/c mice (22-28 g body weight) were housed in individually ventilated cages at a 12 hour light/dark cycle. A single dose of adriamycin (10 mg/kg body weight; BW) was intravenously injected from tail vein to induce nephropathy, while control mice received saline. Hyperoside groups received intraperitoneal injections of 20 mg/kg hyperoside once every day for 2 weeks. Mice were sacrificed at day 15, and kidneys were harvested for various experiments. The experimental procedures and housing conditions were approved by the Nanjing Medical University Institutional Animal Care and Use Committee.

### Cell culture

Conditionally immortalized human podocytes were kindly provided by Dr. Moin A. Saleem (University of Bristol, Bristol, UK) and cultured and differentiated as described previously [43]. Briefly, podocytes were cultured with RPMI 1640 medium (Gibco, MD, catalog number 11875-093) supplemented with 10% fetal bovine serum (Gibco, MD, catalog number 10099-141) and insulin-transferrin-selenite supplement (Life Technologies, CA, catalog number 41400045). Podocytes were maintained in non-permissive conditions at 37 °C for 14 days to induce differentiation and then used for the experiments.

### Renal histology and electron microscopy

Histological and electron microscopic examinations were performed according to the protocol as described previously [17]. Kidney sections (3 μm) were stained with periodic acid–Schiff (PAS) and then examined by light microscopy. The samples subjected to electron microscopic examination were performed using a confocal microscope (Carl Zeiss, Germany).

### 24-hour urinary albumin excretion

Albumin concentrations in the urine were determined using mouse albumin ELISA kits according to the manufacturer’s directions (Abcam, Cambridge, MA, catalog number ab108792).

### Western blots

Protein extracts were prepared from renal cortical tissue samples and cells using a standard method. Briefly, 30 μg protein extracts were subjected to 10% SDS-PAGE and transferred to PVDF membranes. Immunoblotting was performed with primary antibodies. The blots were developed by Pierce enhanced chemiluminescence (ECL, Thermo Fisher Scientific, Rockford, IL, USA, catalog number 32106). Densitometric analysis was performed using Quantity One Software (Bio-Rad, Hercules, CA, USA).

### Immunohistochemistry

Immunohistochemical analyses were performed on paraffin-embedded renal sections (3 μm). The sections were incubated overnight at 4 °C with primary antibodies for podocin. After washing with PBS, the secondary antibody was applied, and the signal was visualized using an avidin–biotin complex (ABC) kit (Santa Cruz Biotechnology, catalog number sc-516216).

### Mitochondrial DNA (mtDNA) copy number

Total DNA from kidney cortex and cultured podocytes were extracted and detected by real-time RT-PCR as described previously [17]. Relative amounts of mtDNA copy number were normalized to 18S ribosomal RNA levels encoded by the nuclear DNA. The primer pairs used were: mtDNA: forward 5’-ATC CTC CCA GGA TTT GGA AT-3’, reverse 5’-ACC GGT AGG AAT TGC GAT AA-3’, 18S rRNA: forward 5’-TTC GGA ACT GAG GCC ATG ATT-3’, reverse 5’-TTT CGC TCT GGT CCG TCT TG-3’.

### Measurement of ROS generation

For *in vivo* examination, kidneys were isolated and frozen in optimal cutting temperature medium. Series of 5 μm thick slices were sectioned from the kidney immediately. Then the slices were incubated with DHE (10 μM) in PBS for 30 minutes. Results were visualized using a confocal microscope (Carl Zeiss, Germany). For *in vitro* study, superoxide levels were assessed by incubating cells with MitoSOX (5 μM) and H_2_O_2_ production was detected by DCFDA (10 μM) staining as previously described [44].

### Drp1 translocation

The mitochondria fraction was isolated from kidney cortex using a mitochondria isolation kit (Thermo Fisher Scientific, Rockford, IL, catalog number 89801) according to the protocol. Western blot was performed to study Drp1 translocation from cytosol to mitochondria. VDAC served as a mitochondrial marker and was used as a protein loading control.

### F-actin immunofluorescence staining

After treatment, podocytes were fixed in 3.7% paraformaldehyde and then stained with Acti-stain™ 488 phalloidin (Cytoskeleton, Inc., USA, catalog number PHDG1) for 30 min at room temperature in darkness. Nuclei were stained with DAPI (Invitrogen, UK, catalog number 62248) for 5 min. Fluorescent images were obtained by using a confocal microscope (Carl Zeiss, Germany). For quantification, 10 random images for each sample were captured and the percentage of cells with disorganized F-actin to total cells for each field was evaluated according to a previous study [45].

### Apoptosis

Podocyte apoptosis was quantified by flow cytometry using annexin-V-fluorescein isothiocyanate (FITC)/propidium iodide (PI) staining following the manufacturer’s protocols (BD Biosciences, San Diego, CA, catalog number 556547). Briefly, after treatment, each group of podocytes was harvested by trypsinization, washed twice with cold PBS, and then resuspended with 100 ul binding buffer (10 mM HEPES, 140 mM NaCl and 2.5 mM CaCl2, pH 7.4) followed by incubation with 5 ul of Annexin V (conjugated with FITC) and 5 ul of PI in the dark for 10 min. The percentage of total apoptotic cells (Q2+Q4) was calculated and shown in the histogram.

### Mitochondrial membrane potential and mitochondrial morphology

As previously described [9], mitochondrial membrane potential was detected with the 5,5’,6,6’-tetrachloro-1,1’,3,3’-tetraethyl-benzimidazolyl carbocyanine iodide (JC-1) fluorescence dye (Molecular Probe, Eugene, OR, catalog number T3168) and podocytes were stained with 100 nM MitoTracker Red (Molecular Probes, Eugene, OR, catalog number M7512) for visualization and quantification of mitochondrial fission.

### Statistical analysis

The results of the statistical analyses were expressed as the mean ± SEM. All experiments. Statistical analysis was performed by one way-ANOVA and Bonferroni tests. The value of *P*<0.05 was inferred as the threshold for significance.
